# The Contribution of Primary Auditory Cortex to Auditory Categorization in Behaving Monkeys

**DOI:** 10.3389/fnins.2018.00601

**Published:** 2018-08-29

**Authors:** Kate L. Christison-Lagay, Yale E. Cohen

**Affiliations:** ^1^Neuroscience Graduate Group, Perelman School of Medicine, University of Pennsylvania, Philadelphia, PA, United States; ^2^Departments of Otorhinolaryngology, Neuroscience, and Bioengineering, University of Pennsylvania, Philadelphia, PA, United States

**Keywords:** primary auditory cortex, categorization, perception, rhesus macaque, ventral stream

## Abstract

The specific contribution of core auditory cortex to auditory perception –such as categorization– remains controversial. To identify a contribution of the primary auditory cortex (A1) to perception, we recorded A1 activity while monkeys reported whether a temporal sequence of tone bursts was heard as having a “small” or “large” frequency difference. We found that A1 had frequency-tuned responses that habituated, independent of frequency content, as this auditory sequence unfolded over time. We also found that A1 firing rate was modulated by the monkeys’ reports of “small” and “large” frequency differences; this modulation correlated with their behavioral performance. These findings are consistent with the hypothesis that A1 contributes to the processes underlying auditory categorization.

## Introduction

A fundamental goal of the auditory system is to transform acoustic stimuli into discrete perceptual representations (i.e., sounds) (Griffiths and Warren, 2004; Bizley and Cohen, 2013). Auditory perception is thought to be mediated by the neuronal mechanisms occurring in the “ventral" auditory pathway (Romanski et al., 1999; Rauschecker and Tian, 2000; Romanski and Averbeck, 2009; Hackett, 2011; Bizley and Cohen, 2013). In rhesus monkeys, this pathway begins in the anterolateral belt region of the auditory cortex, which receives input from core auditory cortex (including the primary auditory cortex; A1) and the middle lateral belt region of the auditory cortex. The anterolateral belt projects directly and indirectly, via the parabelt, to the ventrolateral prefrontal cortex.

Whereas it is generally thought that the ventral pathway has a critical role in auditory perception, the contributions of each region of this pathway to perception have yet to be fully identified (Rauschecker and Scott, 2009; Romanski and Averbeck, 2009; Hackett, 2011; Giordano et al., 2012; Rauschecker, 2012; Bizley and Cohen, 2013). In particular, the specific contributions of the core auditory cortex to perception remain controversial (Binder et al., 2004; Gutschalk et al., 2005; Lemus et al., 2009; Tsunada et al., 2011; Mesgarani and Chang, 2012; Niwa et al., 2012a,b, 2013; Bizley et al., 2013).

To further elucidate a contribution of core auditory cortex to auditory perception, we tested A1’s role in a categorization task in which rhesus monkeys reported whether the frequency difference between two interleaved sequences of tone bursts was “small” or “large.” This stimulus is akin to that used in human streaming studies in which subjects report “one stream” or “two streams.” We titrated task difficulty by changing the frequency difference between the two tone-burst sequences.

We found that, as this auditory sequence unfolded over time, A1 neurons had frequency-tuned responses that habituated, independent of the frequency difference between the tone-burst sequences. Further, we found that A1 firing rate was modulated by the monkeys’ reports of “small” and “large.” Importantly, the monkeys’ behavioral performance positively correlated with this choice-dependent neuronal modulation. These findings provide evidence that A1 activity contributes to the neuronal mechanisms underlying auditory categorization.

## Experimental Procedures

This study was carried out in accordance with the principles and recommendations of the NIH Guide for the Care and Use of Laboratory Animals and by the University of Pennsylvania Institutional Animal Care and Use Committee. The protocol was approved by the University of Pennsylvania Institutional Animal Care and Use Committee. All surgical procedures were conducted under general anesthesia, using aseptic surgical techniques.

### Experimental Chamber

As we reported recently (Christison-Lagay et al., 2017), behavioral training and recording sessions were conducted in a RF-shielded, darkened room with sound-absorbing walls. During each session, a monkey (*Macaca mulatta*; monkey H or monkey S, both male and ages 16 and 12, respectively) was seated in a primate chair in the center of the room. A calibrated speaker (model MSP7, Yamaha) was placed in front of the monkey at a distance of 1.5 m and at eye level. The monkey moved a joystick, which was attached to his chair, with their right hand to indicate their behavioral report. We synthesized auditory stimuli with Matlab (The MathWorks Inc., Natick, MA, United States) and the RX6 digital-signal-processing platform (TDT Inc.) and were transduced by the Yamaha speaker.

### Identification of A1

A1’s anatomical location on the surface of the superior temporal gyrus was identified using MRI images and the Brainsight (Rogue Technologies) software package (**Figure 3A**; monkey H: right hemisphere; monkey S: left hemisphere). A1 was further defined by its frequency-response properties (see section Auditory paradigms and stimuli) (Recanzone et al., 2000; Rauschecker and Tian, 2004; Kajikawa et al., 2005, 2011; Kusmierek and Rauschecker, 2009; Christison-Lagay et al., 2017).

### Auditory Paradigms and Stimuli

Similar to our previous study (Christison-Lagay et al., 2017), in the “passive-listening paradigm,” we recorded A1 spiking activity while monkeys listened passively to different frequency tone bursts. From the recorded spiking activity, we calculated the “best frequency” of the A1 recording site. The “category” task tested the ability of a monkey to report whether the frequency difference between two interleaved sequences of tone bursts was “small” or “large” (Christison-Lagay and Cohen, 2014). The best frequency of each recording site was integrated into the category task, as described below.

#### Passive-Listening Paradigm

A monkey listened passively while different frequency tone bursts (100 ms duration with a 5 ms cos^2^ ramp; 65 dB SPL; 400 ms inter-tone-burst interval) were presented in a random order. The frequency of the tone bursts varied randomly between 0.4–4 kHz in one-quarter octave steps. We restricted neuronal analysis to this frequency range because this was the range that our monkeys had experience with the category task. The monkeys did not receive any juice rewards or any other behavioral feedback during this paradigm.

#### Category Task

**FIGURE 1:**
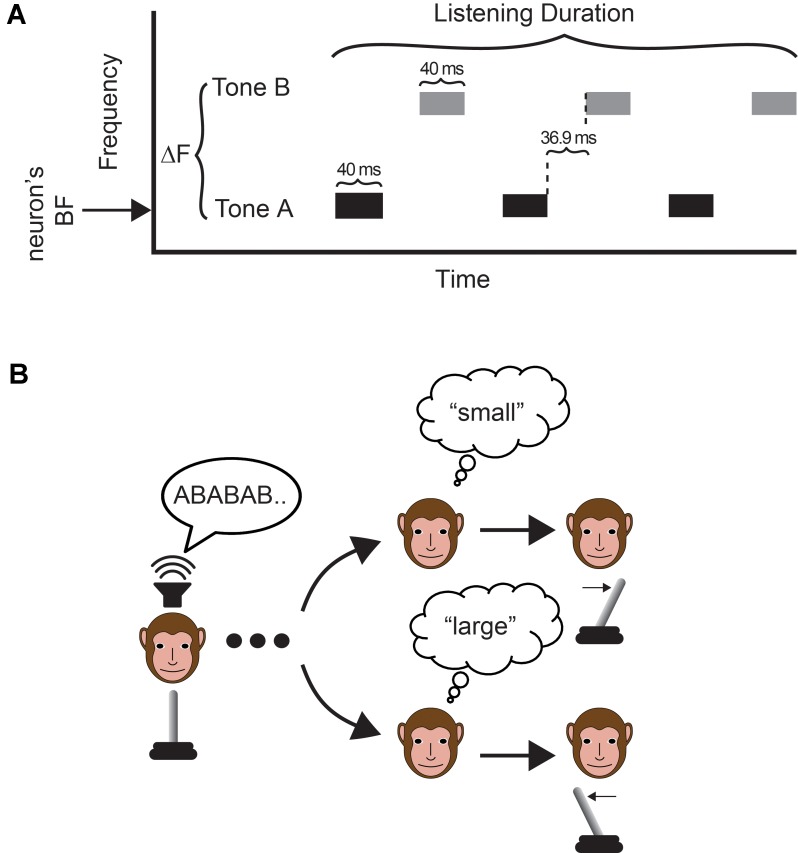
Task and stimulus. **(A)** The auditory stimulus was two temporal sequences of interleaved tone bursts. On a trial-by-trial basis, we varied the frequency difference (ΔF) between the tone-A and tone-B sequences and the duration of the auditory stimulus (listening time). In the tone-A sequence, the frequency of the tone bursts was always set to the recording site’s best frequency. **(B)** The monkey indicated his choice by moving a joystick to the right to report a “small” frequency difference or to the left to report a “large frequency difference”. The monkey made his report following offset of the auditory stimulus.

The category task was a single-interval, two-alternative-forced-choice discrimination task that required a monkey to report whether the frequency difference between two interleaved sequences of tone bursts was “small” or “large”. Five hundred millisecond after the monkey grasped the joystick, we presented the interleaved temporal sequences of tone bursts, which we refer to as the “tone-A” sequence and the “tone-B” sequence, respectively. Following offset of this auditory stimulus, an LED was illuminated, which signaled the monkey to indicate his behavioral report. The monkey moved the joystick (1) to the right to report “small” differences or (2) to the left to report “large” differences (**Figure 1B**). The monkey could only signal his choice following illumination of the LED; in other words, this was not a reaction-time task. The dynamics of this task were comparable to our previous behavioral report (Christison-Lagay and Cohen, 2014).

The tone bursts (40 ms duration with a 5 ms cos^2^ ramp; 65 dB SPL; 76.9 ms inter-tone-burst-interval between A and B frequency tones; 153.8 ms inter-tone-burst-interval between tone bursts of the same frequency) varied only in their frequency values; see **Figure 1A**. In the tone-A sequence, the frequency of the tone bursts was always set to the recording site’s best frequency (see section Data-Collection Strategy below). In the tone-B sequence, the frequency of the tone bursts was 0.5, 3, 5, or 12 semitones above that of tone-A sequence (i.e., the site’s best frequency). The frequency of tone B and the duration of the tone-burst sequence (mean: 750 ± 150 ms; i.e., “listening time”) varied on a trial-by-trial basis.

Monkeys were trained as described in Christison-Lagay and Cohen (2014). Monkeys were rewarded consistently for reporting a “small” frequency difference for 0.5-semitone trials and for a “large” frequency difference for 12-semitone trials. For the 3- and 5-semitone trials, they were given rewards on 50% of randomly selected trials: because these were intermediate between the two frequency-difference extremes, there was not a “correct” response on such trials. Frequency differences of 0.5- (small) and 12- (large) semitone separations each represented ∼33% of trials; 3- and 5- (intermediate) semitone separations each represented ∼16.7% of trials.

### Neuronal-Recording Methodology

At the beginning of each experimental session, a tungsten microelectrode (∼1.0 MΩ @ 1 kHz; Frederick Haër & Co.) was lowered through a recording chamber and into the brain using a skull-mounted microdrive (MO-95, Narishige). OpenEx (TDT Inc.), Labview (NI Inc.), and Matlab (The Mathworks Inc., Natick, MA, United States) software synchronized behavioral control with stimulus production and data collection. Neuronal signals were sampled at 24 kHz, amplified (RA16PA and RZ2, TDT Inc.), and stored for online and offline analyses. Online spike sorting was conducted using OpenSorter (TDT Inc.).

### Data-Collection Strategy

While the electrode advanced through the brain, we presented broadband noise bursts (duration: 100 ms; 65 dB SPL; 50 ms inter-burst-interval), which served as a “search” stimulus to identify auditory-responsive sites. At each site, we isolated the firing rate of a single neuron and determined its best frequency (see section Passive-Listening Paradigm above for more details). A neuron was “auditory” if its firing rate during tone-burst presentation was significantly (*t*-test, H_0_: no difference in firing rate, *p* < 0.05) greater than its firing rate during a baseline silent period of 400 ms that preceded the tone-burst sequence. “Best frequency” was the frequency that elicited the largest response relative to this baseline period. In those instances, when we recorded multiple neurons from a single site, the site’s (and, hence, each neuron’s) best frequency was the same as that of the aforementioned isolated unit; typically, all of the neurons at a recording site had comparable best frequencies. Next, the monkey participated in trials of the category task; see section Category Task for more information on stimulus design and reward structure.

### Behavioral Analyses

A monkey’s performance was quantified as the probability of reporting a “large” frequency difference between tone A and tone B. This was done on a day-by-day basis (*n* = 71 daily experimental sessions) and yielded a distribution of the daily probabilities of reporting a “large” frequency difference. We then tested whether: (1) this distribution of probability values differed from chance (*t*-test, H_0_: mean value of probability distribution = 0.5, *p <* 0.05) and whether (2) the probability-value distributions for the different frequency differences differed from one another [(directional pairwise comparisons and one-way ANOVA (main factor: semitone difference, H_0_: mean probability values were the same)].

### Neuronal Analyses

Neuronal signals were re-sorted offline into single units using Offline Sorter (Plexon Inc., Dallas, TX, United States). Data are reported as firing rate in 20 ms bins (which moved by 2 ms) and were aligned relative the onset of the tone-burst sequence. Additionally, because each tone-burst sequence had a different listening time (i.e., duration), analyses were restricted to the time period encompassed by the first 10 tone bursts, which captured the majority of the data across all of the recording sessions.

#### ROC Analyses

Two analyses quantified A1 sensitivity during the frequency-discrimination task. For both, we calculated a receiver-operating-characteristic (ROC) curve (Green and Swets, 1966). The area under this curve describes the probability that an ideal observer can use the spiking activity of an individual neuron to discriminate between two stimuli or between two behavioral conditions.

In a first ROC analysis, we calculated whether this ideal observer could discriminate between the distribution of firing rates elicited by the first tone A and the distributions measured in subsequent time bins. In this analysis, we used data from all trials regardless of the monkeys’ behavioral reports.

In the second analysis, we calculated whether, on a semitone-by-semitone basis, this ideal observer could use firing rate to discriminate between the monkeys’ reports of “small” and “large” frequency differences for the same nominal stimulus (Britten et al., 1996; Purushothaman and Bradley, 2005; Gu et al., 2007). We report the mean ROC values across our population of recorded neurons.

For both analyses, we report the mean ROC values across our population of recorded neurons. A bootstrap randomization procedure calculated the significance of each of these ROC values. This randomization procedure was conducted on each neuron and for both ROC analyses. A mean ROC value was considered significant if it exceeded the 95% confidence interval of this bootstrapped null distribution.

## Results

### Psychophysical Performance

**FIGURE 2:**
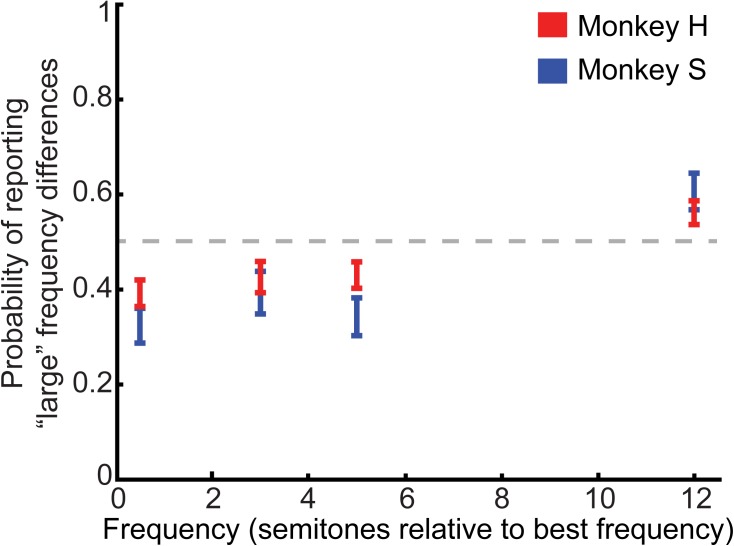
Behavioral performance on the category task. Average psychometric performance for both monkeys is plotted as the probability that the monkeys reported a “large” frequency difference as a function of the frequency difference between the tone-A and tone-B sequences (in semitones). The center of each bar indicates average performance. The length of the bars indicates the 95%-confidence interval. The gray dashed line represents chance performance of reporting a “large” frequency difference (*p* = 0.5).

The behavioral results from 71 experimental sessions (Monkey H: *n* = 46; Monkey S: *n* = 25) are shown in **Figure 2**, which plots the probability that the monkeys reported a “large” frequency difference as a function of the frequency difference between tone A and tone B. Monkeys reliably reported large (12 semitone) and small (0.5 semitone) differences at levels different from chance (*t*-test, H_0_: mean value of probability distribution equal to 0.5, *p* < 0.05); directional *t*-tests confirmed that monkeys reported 12-semitone differences as “large” (one-tailed *t*-test, H_0_: mean value of probability distribution equal to or less than 0.5, *p <* 0.025), and 0.5-semitone differences as “small” (one-tailed *t*-test, H_0_: mean value of probability distribution equal to or greater than 0.5, *p <* 0.025). Pairwise comparisons between 3-semitone trials to 0.5 and 12-semitone trials revealed that the monkeys were more likely to report a “small” difference for 3-semitone trials compared to 12-semitone trials but were more likely to report a “large” difference for 3-semitone trials compared to 0.5-semitone trials (3 vs. 12 semitones: *t*-test, H_0_: mean value of probability distributions for 12 and 3 semitones were equal, *p <* 0.05; one-tailed *t*-test, H_0_: mean value of probability distribution of 3-semitone trials was equal to or greater than the mean value of probability distribution of 12-semitone trials, *p <* 0.025; 3 vs. 0.5 semitones: *t*-test, H_0_: mean value of probability distributions for 0.5 and 3 semitones were equal, *p <* 0.05; one-tailed *t*-test, H_0_: mean value of probability distribution of 3-semitone trials was equal to or less than the mean value of probability distribution of 0.5-semitone trials, *p <* 0.05). Similarly, 5-semitone trials were more likely to be reported as “small” than 12-semitone trials but were more likely to be reported as “large” than 0.5-semitone trials (5 vs. 12 semitones: *t*-test, H_0_: mean value of probability distributions for 12 and 5 semitones were equal, *p <* 0.05; one-tailed *t*-test, H_0_: mean value of probability distribution of 5-semitone trials was equal to or greater than the mean value of probability distribution of 12-semitone trials, *p* < 0.025; 5 vs. 0.5 semitones: *t*-test, H_0_: mean value of probability distributions for 0.5 and 5 semitones were equal, *p <* 0.05; one-tailed *t*-test, H_0_: mean value of probability distribution of 5-semitone trials was equal to or less than the mean value of probability distribution of 0.5-semitone trials, *p <* 0.025). Finally, an ANOVA indicated that the mean probability of reporting a “large” frequency difference differed across semitone conditions (one-way ANOVA, main factor: semitone difference: H_0_: mean probability values were not different from one another, *p <* 0.05). We could not identify any reliable differences between the two monkeys’ performance.

Overall, these findings indicate that the monkeys’ behavioral reports covaried as the frequency difference between the two tone-burst sequences increased.

### Recording-Site Localization

**FIGURE 3:**
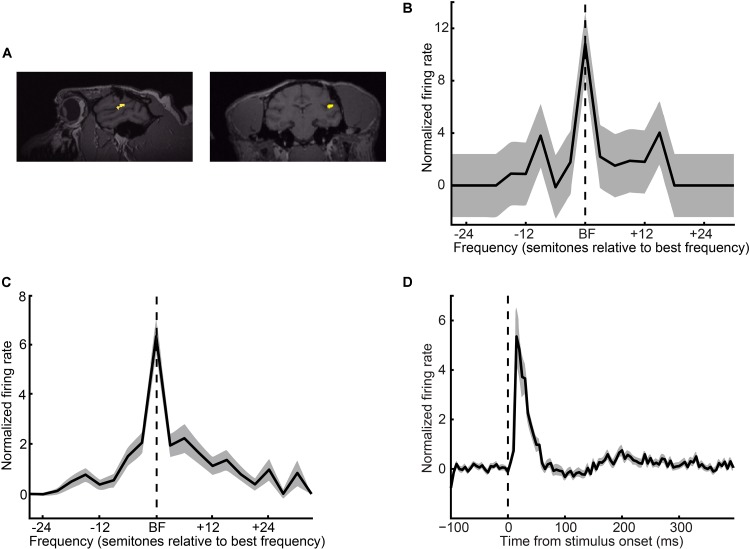
Recording locations and A1 response properties. **(A)** Sagittal and coronal MRI sections of monkey H’s brain at the level of the superior temporal gyrus. The yellow regions indicate the targeted location of A1. **(B)** Single-neuron and **(C)** population frequency-response profiles. These response profiles are plotted relative to a neuron’s best frequency (BF). The vertical dotted line indicates BF. **(D)** Population peristimulus-time histogram. The vertical dotted line indicates tone-burst onset. For panels **(B–D)**, firing rate is normalized relative to a baseline period of silence. Thick lines indicate mean values; shading indicates s.e.m.

We focused on the contribution of A1 (**Figure 3A**) to auditory categorization and perception. We isolated 108 A1 single units (61 from monkey H and 47 from monkey S) and found, as expected, that A1 neurons were frequency tuned (**Figures 3B,C**) with short latency responses (**Figure 3D**). In our population, best frequencies were evenly distributed between 400 and 3940 Hz (median: 1984 Hz). The median Q-value (an index of tuning sharpness; best frequency divided by bandwidth) was 1.05 (25% quartile: 0.41; 75% quartile: 2.5). Median latency (i.e., the first of two or more consecutive time bins that were > 2 s.d. above a 400 ms baseline period of silence) was 15 ms (25% quartile: 10 ms; 75% quartile: 35 ms). This collection of neurophysiological-response properties is consistent with those seen from our group and in earlier studies of A1 (Recanzone et al., 2000; Fu et al., 2004; Kajikawa et al., 2005; Kikuchi et al., 2010; O’Connell et al., 2014; Massoudi et al., 2015; Christison-Lagay et al., 2017) and verifies our targeted recording-site location.

### A1 Spiking Activity Habituates During the Category Task in a Frequency-Independent Manner

**FIGURE 4:**
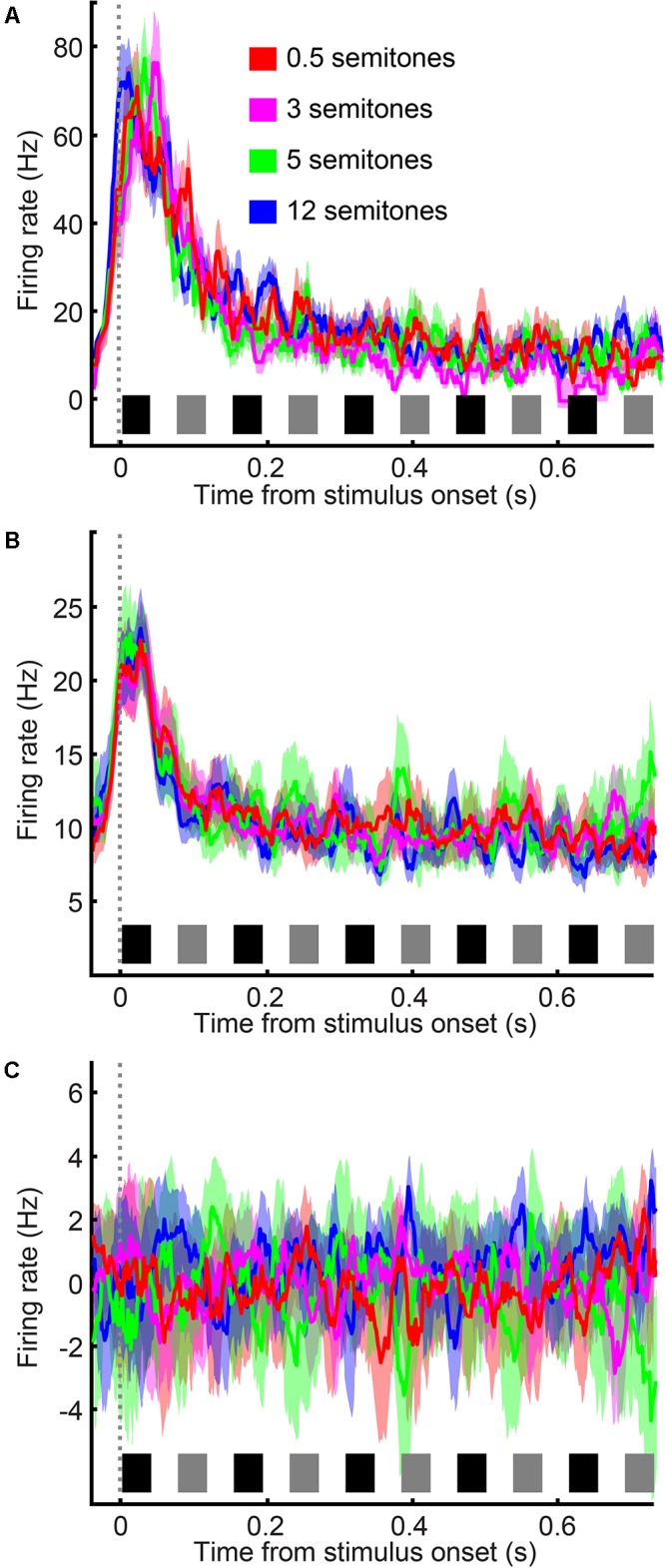
A1 neurons habituate over time in a frequency-independent manner. **(A)** Single-neuron example of A1 firing rate in response to the tone-burst sequence; data are combined from reports of “small” frequency difference and “large” frequency difference. Color corresponds to the frequency difference between the tone-A and tone-B sequences; see legend. Data are aligned relative to tone A_1_, which is first tone burst in the sequence (sliding window of 20 ms that moved in 2 ms increments). **(B)** Population histogram, plotted as in **(A)**. The thick black line indicates the average response across all frequency differences. **(C)** Population histogram in which the mean response across all semitones was subtracted from each neuron’s response as a function of semitone separation. Thick lines indicate mean values; shading indicates s.e.m. The black and gray rectangles above the x-axis show the time course of each tone burst (tone-As and tone-Bs, respectively) in the auditory sequence.

**FIGURE 5:**
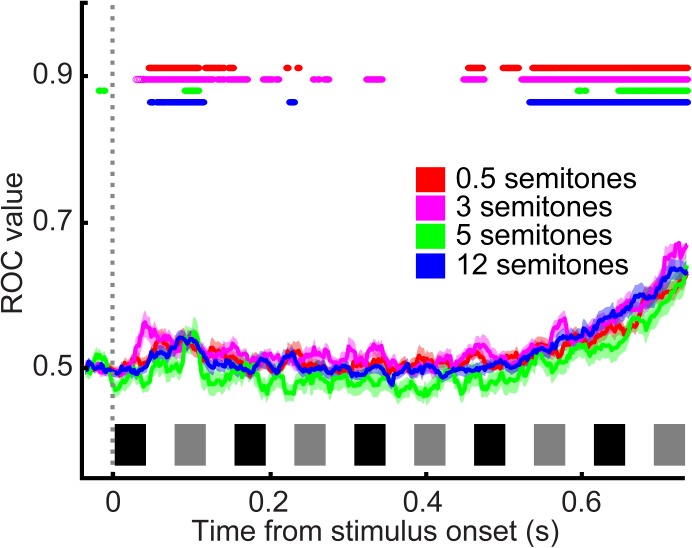
ROC analysis quantifying A1 habituation. ROC values relative to average firing rate elicited by tone A_1_ as a function of time. Data are combined from reports of “small” frequency difference and “large” frequency difference and are aligned relative to tone A_1_ (sliding window of 20 ms that moved in 2 ms increments). Color corresponds to the frequency difference between the tone-A and tone-B sequences; see legend. Thick lines indicate mean values; shading indicates s.e.m. Colored lines at the top of the graph indicate the time when mean ROC values were significantly different than chance (*t*-test, H_0_: ROC value = 0.5, *p* < 0.05; see legend). The black and gray rectangles above the x-axis show the time course of each tone burst (tone-As and tone-Bs, respectively) in the auditory sequence.

During the category task, A1 spiking activity was modulated primarily by the time course of the tone-burst sequence (single neuron: **Figure 4A**; population activity: **Figure 4B**). A1 neurons responded most vigorously to the presentation of tone A_1_ (i.e., the first tone burst in the tone-A sequence, which as a reminder, was at the best frequency of the recording site) and were less responsive to subsequent tone bursts. To quantify this observation, we conducted a running ROC analysis (Green and Swets, 1966) to test how neuronal activity changed as the tone-burst sequence unfolded over time (**Figure 5**). On a neuron-by-neuron basis, we calculated a running ROC (sliding window of 20 ms that moved in 2 ms increments), which compared the average firing rate elicited by tone A_1_ to subsequent firing rates; this analysis used both correct and incorrect trials. An ROC value of 0.5 indicates that an ideal observer could not distinguish between the firing rate elicited by tone A_1_ and the firing rate elicited at any later point in the tone-burst sequence; whereas a value of 1 indicates that this observer could perfectly distinguish between these two firing rates. We found that ROC values generally increased with time, indicating that the firing rate in response to tone A_1_ became increasingly different than subsequent tone-elicited firing rates. In our case, these firing rates decreased (i.e., habituated) over time. This habituation was reported previously in streaming studies that did not have simultaneous behavioral reports (Fishman et al., 2001, 2004; Ulanovsky et al., 2004; Micheyl et al., 2005).

However, unlike these earlier studies, we did not find that the frequency difference between the tone-A and tone-B sequences had any substantial effect on habituation. Indeed, although some individual A1 neurons displayed a small amount of frequency-dependent habituation (e.g., see responses in **Figure 4A**), on average, this dependency was not observed (**Figure 4B**). This is most clearly seen in **Figure 4C**, where we have removed habituation’s mean effect. To remove this effect, we calculated, as a function of time, the mean firing rate across all frequency differences and subtracted this mean firing-rate time course from each neuron’s response as a function of the frequency difference between the tone-A and tone-B sequences. As can be seen, this subtraction procedure indicated that A1 spiking activity was not significantly modulated by the frequency difference between the tone-A and tone-B sequences [1-factor ANOVA (main factor: frequency difference), H_0_: mean firing rate was the same, *p* > 0.05].

### A1 Neurons and Monkeys’ Choices Are Comodulated

**FIGURE 6:**
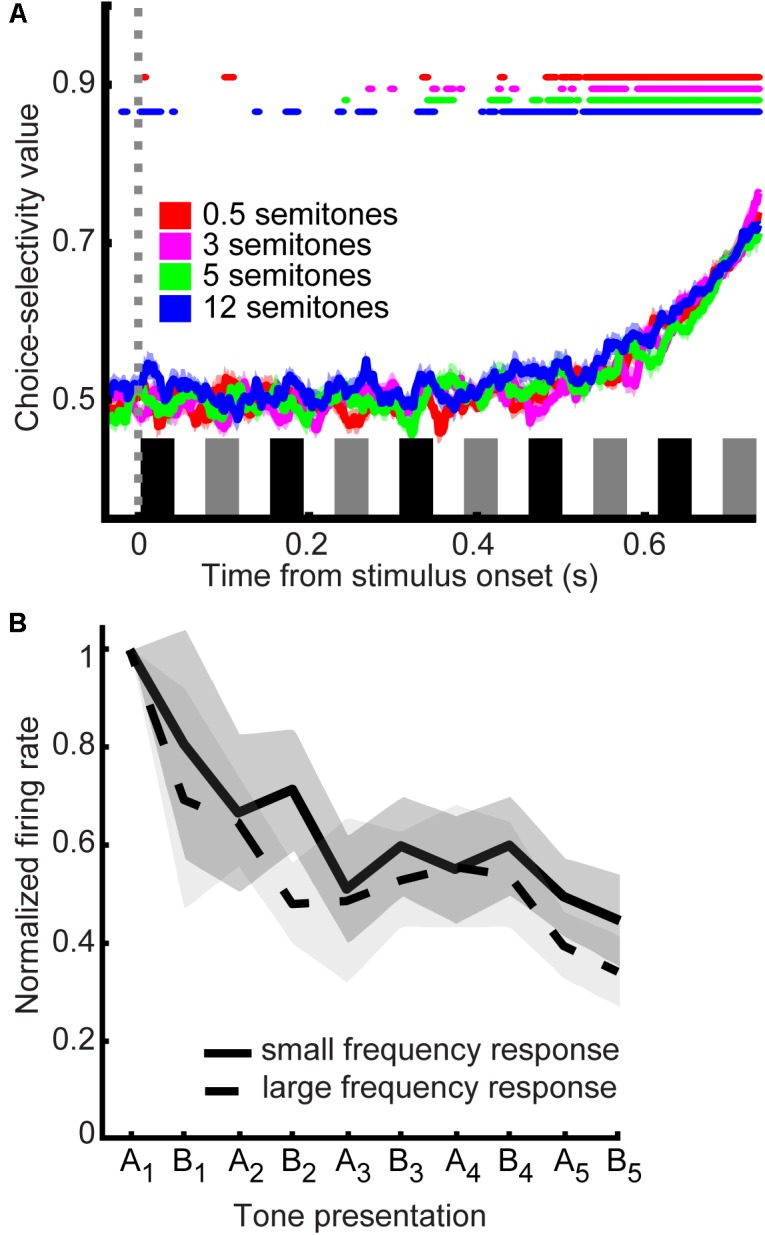
Choice selectivity of A1 neurons. **(A)** Choice probability values as a function of time. Data are aligned relative to tone A_1_ (sliding window of 20 ms that moved in 2 ms increments). Color corresponds to the frequency difference between the tone-A and tone-B sequences; see legend. Thick lines indicate mean values; shading indicates s.e.m. Colored lines at the top of the graph indicate the time when mean choice-probability values were significantly different than chance (*t*-test, H_0_: choice-probability value = 0.5 *p* < 0.05; see legend). The black and gray rectangles above the x-axis show the time course of each tone burst (tone-As and tone-Bs, respectively) in the auditory sequence. **(B)** Response profile as a function of choice. Population response profile of A1 firing rate in response to the acoustic sequence for five semitone trials data are separated by reports of “small frequency difference” and “large frequency difference”. The solid line indicates the average firing rate for reporting a small frequency difference; the dotted line indicates the average firing rate for reporting a large frequency; thick lines indicate mean values; shading indicates s.e.m. Data are aligned relative to each tone burst in the sequence; inter-tone silent period is not shown. The first tone burst is designated as “A_1_”; the second as “B_1_,” the third as “A_2_” etc.

To test the relationship between A1 activity and the monkeys’ decisions, we conducted a second ROC analysis that quantified the “choice selectivity” of our neuronal population. Specifically, this analysis quantified ability of an ROC-based ideal observer to use spiking activity to discriminate between “small” and “large” choices for nominally identical stimuli (**Figure 6A**). A value of 0.5 indicates that an ideal observer could not discern the monkeys’ choices from an A1 neuron’s firing rate, whereas a value of 1.0 indicates that this observer could perfectly discern the monkeys’ choices. We calculated a running ROC analysis (sliding window of 20 ms that moved in 2 ms increments), independently for the 0.5-, 3-, 5-, and 12-semitone trials. For the first 0.4 s of the tone-burst sequence, the mean values were not consistently different than chance (*t*-test, H_0_: choice-probability value = 0.5 *p* > 0.05). However, with longer listening times, these ROC values increased. Essentially, choice-probability values across all semitone trials became consistently significant (*t*-test, H_0_: choice-probability value = 0.5, *p* < 0.05) after ∼0.5 s of listening time and then continued to increase to ∼0.7 with additional listening.

At least part of the basis for these increasing choice-probability values is differences in firing rate over time, which can be very marked in some neurons. **Figure 6B** shows the activity for “small” vs. “large” frequency reports across neurons for the 5-semitone trials (normalized to the firing rate elicited by the first presentation of the A tone). We show the 5-semitone data as a representative exemplar and because 5 semitone trials are approximately equally divided between reports of “large” and “small” frequency differences. As can be seen, the mean begins to differentiate later in the trial, which results in the pattern of increasing choice-probability values over time as seen in **Figure 6A**.

**FIGURE 7:**
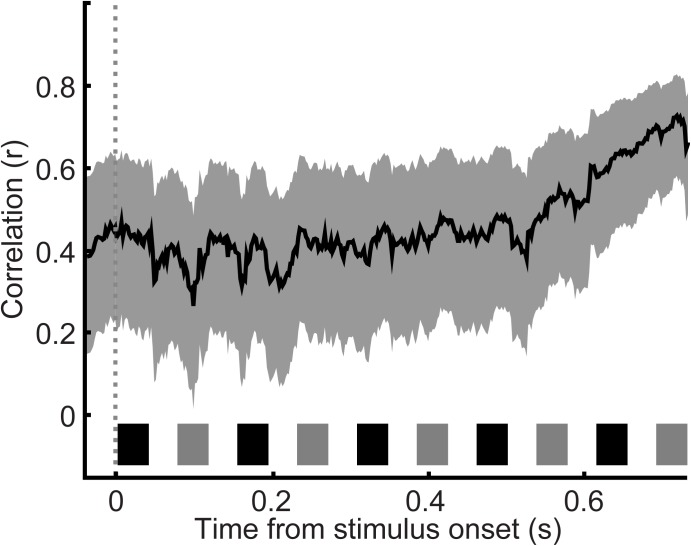
Correlation between choice probability and concurrently measured behavioral performance. Behavioral performance–defined as the overall performance on 0.5 and 12 semitone separation trials during the recording session–was correlated with choice probability values (as calculated in **Figure 6A**) as a function of time. Data are aligned relative to tone A_1_ (sliding window of 20 ms that moved in 2 ms increments). Thick lines indicate Pearson correlation r values; shading indicates the 95%-confidence interval of the Pearson *r*-values. The black and gray rectangles above the x-axis show the time course of each tone burst (tone-As and tone-Bs, respectively) in the auditory sequence.

Finally, to gain further insight into the relationship between choice probability and the monkey’s behavior, we analyzed, as a function of time, the session-by-session relationship between these ROC values and concurrently measured behavioral performance (**Figure 7**). Our index of behavior was overall performance on the 0.5- and 12-semitone trials (i.e., probability of correct “small” and “large” reports) during a particular session. This correlation was significant (Pearson correlation coefficient, *p* < 0.05) throughout the entire listening period.

## Discussion

The psychophysical principles that underlie a listener’s perceptual organization of their acoustic environment into distinct auditory streams are well-described (Bregman, 1990; Griffiths and Warren, 2004; Oxenham, 2008; Winkler et al., 2009; Shamma et al., 2011, 2013; Bizley and Cohen, 2013; Middlebrooks, 2013), but the relationship between behavioral reports of streaming and single-neuron cortical activity has not been fully elucidated. Here, although our monkeys were tasked with making “small” vs. “large” reports, our stimulus is comparable to those used in streaming tasks when listeners make reports of “one stream” and “two streams.” This included using intermediate frequency differences, which in humans elicit responses of either one or two streams (Bregman, 1990). Indeed, earlier studies suggest that monkeys show many hallmarks of auditory streaming (Selezneva et al., 2012; Christison-Lagay and Cohen, 2014). In our earlier behavioral study (which used the same animals and same experimental stimuli as in the current study) (Christison-Lagay and Cohen, 2014), we included three control conditions that support that monkeys stream the stimuli used in the current paradigm. First, the monkeys were more likely to report “large” frequency differences with longer listening times (Christison-Lagay and Cohen, 2014), similar to human listeners who are also more likely to report two streams with longer listening times (Micheyl et al., 2005) and consistent with the idea that one’s perception of two streams “builds up” over time (Micheyl et al., 2005; Haywood and Roberts, 2013). Second, similar to human listeners (Elhilali et al., 2009; Micheyl et al., 2013), when the tone bursts were presented simultaneously (vs. asynchronously as shown in **Figure 1A**) and the frequency difference was large (which normally elicits reports of “two auditory streams”), the monkeys’ reports were biased toward those of when they had heard a “small” frequency difference (Christison-Lagay and Cohen, 2014). Again, although we cannot know the monkeys’ subjective perceptions, their pattern of behavioral reports – although clearly with overall poorer performance – is consistent with those of human reports during streaming tasks.

It is important to note that there was a minor decrease in peak performance between the current and previous studies (Christison-Lagay and Cohen, 2014), even though the same monkeys were used in both studies. This can be attributed to periods of significantly higher performance in the behavior-only study, analyses of which were presented in our previous study. We did not find a robust difference in performance in the behavior during the behavior-only sessions and the recording sessions.

One further caveat is important to note when comparing our current study to previous streaming studies: the current paradigm presented stimuli at a faster rate than many previous studies (Bregman, 1990; Griffiths and Warren, 2004; Oxenham, 2008; Shamma et al., 2011, 2013; Bizley and Cohen, 2013; Middlebrooks, 2013). This may partially account for inability to clearly disambiguate the separate responses to the different frequency tones (Fishman et al., 2001, 2004; Micheyl et al., 2005).

Further, because of this fast presentation rate, we are not able to speak to how much of the observed habituation was due to stimulus-specific adaptation or some other mechanism: we observed habituation to presentations of the B-frequency tones, independent of the frequency difference between the A- and B-frequency tones. If all of the habituation were due to stimulus-specific adaptation, then one might expect that when tone B’s frequency was close to tone A’s frequency (e.g., 0.5 semitones higher), that a greater degree of adaptation would be observed to the B frequency than when B’s frequency was much different than A’s (e.g., 12 semitones higher). However, as shown in **Figures 4C, 5**, we do not find this. In future studies, it would be important to vary the relationship between the frequency of tone A and a neuron’s best frequency, as well as modulating tone B’s frequency relative to the frequency of tone A.

Our interpretation of the neuronal data does not hinge on whether or not the monkeys were, in fact, reporting their streaming percepts or rather reporting “small” and “large” frequency differences: regardless of either interpretation, the current data are important for understanding scene analysis because frequency differences are a primary way by which the auditory system segments auditory stimuli into discrete auditory streams (Bregman, 1990). However, it is also important to consider them through the lens of other auditory categorization tasks. Many studies have used complex sounds, and have found that categorization emerges later in auditory processing (Steinschneider et al., 2003; Gifford et al., 2005; Cohen et al., 2006; Russ et al., 2007, 2008; Lee et al., 2009; Tsunada et al., 2011; Nieder, 2012). Relatively fewer studies have looked at categorization of low-level auditory stimuli, such as tones. However, several of these studies have found results consistent with our reported results: A1 may have activity related to category at the single-neuron level (Selezneva et al., 2006, 2017), population level (Ohl et al., 2001), or within subpopulations of neurons (Jaramillo et al., 2014). It is important to note that although our task is arguably categorical, our monkeys reported identical stimuli as belonging to different categories on a trial-by-trial basis; moreover, the neurons reflected these trial-by-trial categorical judgments.

Regardless of whether monkeys reported “small” or “large” frequency difference or stream percepts, our main neurophysiological finding is of interest: A1 activity was modulated by the monkeys’ perceptual choices. As mentioned previously, the specific contribution of the auditory cortex to perception and choice behavior is controversial. Consistent with our current findings (**Figure 6A**), recent studies (Niwa et al., 2012b, 2013; Bizley et al., 2013) have demonstrated that A1 has choice-related activity. However, the contribution of A1 to perception resists a simple story: other previous work, including work from our group (Lemus et al., 2009; Tsunada et al., 2011, 2016), has shown that neurons in the auditory cortex are not reliably modulated by choice. There are two notable corollaries to this latter point within work for our group: the anterolateral belt of the auditory cortex does appear to causally contribute to certain types of auditory decisions (Tsunada et al., 2016), and choice-related activity has been found in population-level activity of A1 neurons (though not reliably at the single neuron basis) (Christison-Lagay et al., 2017).

We cannot reconcile these different sets of findings, but we hypothesize that it may relate to the specific demands of the auditory decision. For those studies that demonstrated significant choice-related activity in A1 (current findings **Figure 6A**; Niwa et al., 2012a,b, 2013; Bizley et al., 2013), the animal listeners were asked to make a decision about a relatively low-level perceptual attribute (e.g., pitch and amplitude modulation). Because this attribute may be represented directly in an A1 neuron’s firing rate, A1 activity may be able to encode the sensory evidence for these types of decisions. In contrast, it is possible that monkeys were required to make a higher-level decision about a perceptual attribute that may not be encoded directly in a neuron’s firing rate in studies that did not identify choice-related activity in the auditory cortex (Binder et al., 2004; Lemus et al., 2009; Tsunada et al., 2011, 2016). For such decisions, it is feasible that only later regions of the ventral auditory pathway can encode the proper sensory evidence. Thus, the function of each cortical region of the ventral pathway may be modulated by the specific stimuli, nature, and demands of an auditory decision (Bizley and Cohen, 2013; Bizley et al., 2013; Niwa et al., 2013). Additionally, even in studies that did not find perceptual or choice-related activity in early auditory areas, it is possible that such information was encoded at the population-level instead of at single-neuron level (Christison-Lagay et al., 2017).

Finally, the presence of A1 choice-related activity does not imply that it is part of a feedforward process that contributes causally to an eventual auditory decision (Nienborg and Cumming, 2009; Niwa et al., 2013; Tsunada et al., 2016). This activity may arise via feedback from regions of the ventral auditory pathway that represent the auditory decision (Nienborg and Cumming, 2009). Future work should focus on using response-time tasks (Stuttgen et al., 2011; Tsunada et al., 2016) to identify the temporal window of the auditory decision in order to differentiate between these feedforward vs. feedback alternates (Cohen and Newsome, 2009; Nienborg and Cumming, 2009).

## Author Contributions

KC-L and YC conceived and designed the research, interpreted the results of experiments, drafted, edited, revised the manuscript, and approved final version of the manuscript. KC-L performed the experiments, analyzed the data, and prepared the figures.

## Conflict of Interest Statement

The authors declare that the research was conducted in the absence of any commercial or financial relationships that could be construed as a potential conflict of interest.
